# Editorial: Reviews in cancer imaging and image-directed interventions

**DOI:** 10.3389/fonc.2023.1183302

**Published:** 2023-03-16

**Authors:** Omar S. Al-Kadi, Oliver Diaz

**Affiliations:** ^1^ Department of Artificial Intelligence, King Abdullah II School for Information Technology, University of Jordan, Amman, Jordan; ^2^ Departament de Matemàtiques i Informàtica, Universitat de Barcelona, Barcelona, Spain

**Keywords:** cancer imaging, medical image analysis, artificial intelligence, image- guidance, cancer diagnostics, deep learning

This Research Topic is concerned with the application of medical imaging in the diagnosis and treatment of cancer. It includes the use of a range of imaging modalities, such as computed tomography (CT), magnetic resonance imaging (MRI), positron emission tomography (PET), optical coherence tomography (OCT), molecular luminescence spectroscopy, and their applications in detecting and characterizing tumors, as well as guiding interventions such as biopsies and targeted therapies. The reviews in this field aim to provide clinicians with a comprehensive understanding of the latest advances in cancer imaging and image-directed interventions, including the use of artificial intelligence (AI) and other emerging technologies. By keeping up with the latest research, clinicians can improve patient outcomes and enhance the overall quality of cancer care. The editorial article discusses 9 reviews, 7 systematic reviews, 1 mini review articles, covering organs such as liver, brain, lung, prostate, abdominal, and oral and maxillofacial regions.

Starting with the upper most part of the human body, brain cancer is a complex disease that can have a profound impact on individuals, and understanding its importance requires a comprehensive understanding of its causes, symptoms, and potential treatments. Xu et al. discusses the difficulty in diagnosing and managing glioma, and how medical imaging techniques like MRI, PET, and spectral imaging can aid physicians in treatment. The authors highlight the use of AI in medical imaging analysis, specifically in glioma diagnosis and management, such as tumor segmentation and classification, prediction of genetic markers, and treatment response and prognosis. However, the authors note that there are still issues to be solved with AI in clinical applications, such as data management, safety, and ethical and legal considerations. They suggest that interdisciplinary teamworks between clinicians and researchers are necessary to solve these issues in the future. Also, the meta-analysis in Jiang et al. evaluates the effectiveness of radiomics using non-enhanced computed tomography (NCCT) in predicting hematoma expansion in patients with spontaneous intracerebral hemorrhage. Ten articles comprising 1,525 subjects were analyzed, and the radiomics model showed an Area under the curve (AUC) of 0.80. Results revealed that the radiomics model outperformed most of the NCCT biomarkers in predicting hematoma expansion. The study suggests that the radiomics approach has the potential to predict hematoma expansion and is recommended over NCCT biomarkers. However, standardization of the radiomics pipeline is necessary for further clinical implementation.

Continuing in the upper part of the body, particularly the oral and maxillofacial region, Zhu et al. evaluated the accuracy of deep learning (DL) using the convolutional neural network VGGNet model in distinguishing benign and malignant thyroid nodules, based on ultrasound images. A total of 11 studies were included in the meta-analysis, and the overall estimates of sensitivity and specificity were 0.87 and 0.85, respectively. The results suggest that DL using the VGGNet model with ultrasound images performed good diagnostic efficacy in distinguishing benign and malignant thyroid nodules. Also, Wu et al. aimed to evaluate the diagnostic value of elastosonography for detecting salivary gland tumors and compare it to conventional ultrasound. This review analyzed 16 studies with a total of 1,105 patients and found that elastosonography had a pooled sensitivity of 0.73 and specificity of 0.64 for differentiating between benign and malignant tumors, with an AUC of 0.82. The study also found that quantitative or semi-quantitative elastosonography performed better than the qualitative one. The authors concluded that elastosonography could be considered a supplementary diagnostic technology to conventional ultrasound for detecting salivary gland tumors.

Moving down to the thoracic cavity, the review article by Van De Stadt et al. discuss the need for alternative biomarkers to predict tumor response to EGFR tyrosine kinase inhibitor therapy in NSCLC, highlighting the limitations of biopsies. PET studies using EGFR TKI-based tracers have shown promise in identifying EGFR mutational status and as a potential biomarker for tumor response. The article discusses currently investigated EGFR-directed PET biomarkers, their development process, and the advances, challenges, and opportunities for EGFR PET biomarkers to be used in routine clinical practice. Another work (Liang et al. **)** analyzed 19 original studies involving 2,444 patients and 3,012 subsolid pulmonary nodules (SSNs). They identified 18 clinical and CT features that correlated with SSN growth, including independent risk factors such as male sex, history of lung cancer, nodule size > 10 mm, nodule consistency, and age > 65 years. These findings can aid in establishing risk-based follow-up management strategies for SSN patients.

The abdominal cavity is the largest hollow space in the body, and detecting cancer in this area is crucial due to the vital organs located there, that are essential for proper body function. Moreover, detecting cancer in the abdominal cavity is challenging, particularly during its early stages. In Liang et al., radiomics-based MRI are evaluated for predicting microvascular invasion (MVI) in hepatocellular carcinoma (HCC) through a review of 15 studies involving 981 patients. The results show that radiomics-based MRI has high accuracy in predicting MVI in HCC, with a pooled sensitivity of 0.79, specificity of 0.81, and AUC of 0.87. Though there is heterogeneity among studies, sensitivity analysis supports the reliability of the results. The paper highlights the development of AI-based tools for liver cancer treatment. The use of interventional therapy for liver cancer is presented in Ren et al.. These tools can assist clinicians in making more precise diagnoses, treatment plans, and preventative measures for liver cancer patients, leading to more rational and personalized care. The article emphasizes that AI is bringing disruptive changes to the traditional medical model. Another systematic review article **(** Zhou et al. **)** compare two categorization systems used to diagnose hepatocellular carcinomas (HCCs) and determine their diagnostic performance. The systems compared were Contrast-enhanced ultrasound (CEUS) LI-RADS and CT/MRI LI-RADS. The study included 43 studies, and the results showed that CEUS LR-5 and CT/MRI LR-5 had similar diagnostic performance for HCCs, while CEUS LR-M had a higher proportion of HCCs and a lower proportion of non-HCC malignancies than CT/MRI LR-M. The study also found that CEUS LR-3 had a lower risk of HCCs than CT/MRI LR-3.

Moving to the undersurface of the right lobe of the liver, the work by Li et al. is interested in the application of DL in the imaging assessment of bladder cancer (BCa). DL has shown great potential in solving medical problems, particularly in the field of medical imaging. The authors provide an overview of current DL approaches used for bladder segmentation and how it helps in the diagnosis, staging, and treatment management of BCa. Also, Liu et al. discusses the application of DL in the diagnosis of gastrointestinal subepithelial lesions (SELs) using endoscopic ultrasonography (EUS). The study found that AI-assisted EUS is a promising and reliable method for distinguishing SELs, with excellent diagnostic performance, and is superior to EUS by experts. The authors recommend conducting more multicenter cohort and prospective studies to further develop AI-assisted real-time diagnostic systems and validate the superiority of AI systems. Furthermore, Liu et al. develop advanced ultrasound examination modes for diagnosing prostate cancer (PCa), including micro-Doppler, computerized-transrectal ultrasound, elastography, contrast-enhanced ultrasound, and microultrasound, collectively referred to as multiparameter ultrasound (mp-US). The combination of two or more of these modes can provide complementary information to multiparameter magnetic resonance imaging (mp-MRI) for diagnosing PCa. The authors suggest that mp-US has great potential as an imaging method for the diagnosis of PCa.

On the use of molecular luminescence for cancer imaging, the work by Chen et al. discusses the limitations of current surgical techniques for treating bone and soft tissue sarcoma and the potential for intraoperative fluorescence imaging to assist surgeons in determining tumor boundaries during surgery. The review considers the use of fluorescence imaging technology in clinical studies and assesses the potential of this technique to improve the accuracy of surgical resection. It suggests that intraoperative fluorescence imaging is a safe and straightforward technique that does not add any additional time to the surgery and has promising applications for the treatment of bone and soft tissue sarcoma. In the same realm, the work by Yang et al. shows recent advances in OCT modality, and its application in oncological diagnosis and treatment. The review highlights how OCT imaging can be used to detect and diagnose superficial and deep tumors in different types of cancers such as skin, gastrointestinal, brain, breast, bladder, and lung cancers, and how it can monitor tumor responses to treatments. Furthermore, the work in Yi et al. review molecular imaging techniques for cancer diagnosis and treatment, focusing on small-molecule inhibitors as cancer target probes. They summarize the structural designs of affinity probes based on small-molecule inhibitors and their impact on affinity and pharmacokinetics. The authors present clinical examples and provide insights for future research and clinical translations.

Others **(** Deng et al. **)** focused on the important role of NR4A1, a nuclear subfamily 4 receptor, in regulating metabolism in various cancers including melanoma, colorectal cancer, breast cancer, and hepatocellular cancer. NR4A1 has been found to mediate glycolysis, fatty acid synthesis, glutamine metabolism, and tumor immunity in cancer cells. The review suggests that regulating NR4A1 with novel ligands could be a promising approach to alter metabolism signaling pathways in cancer therapy.

Finally, the use of machine learning in cancer diagnostics, specifically focusing on the benefits of semi-supervised learning (SSL) compared to supervised learning (SL) is presented in Eckardt et al. SSL can use unlabeled samples in addition to labeled data for information abstraction, which allows for more efficient use of available data in cancer diagnostics. The article provides an overview of SSL functionalities and assumptions, and surveys key studies in image-based and non-image-based applications of SSL in cancer care, including histopathology, radiology, radiotherapy, and genomics. The authors highlight recent models and potential pitfalls in SSL study design, and suggest future directions for SSL in oncology.

We hope that this Research Topic will serve as a valuable resource for individuals interested in the important field of Cancer Imaging and Image-directed Interventions. It aims to present the most recent experimental methods used to explore fundamental concepts in Cancer Imaging and Image-directed Interventions, as well as to showcase the latest breakthroughs in the field. Furthermore, this topic underlines important areas for future research while also highlighting new clinical and therapeutic opportunities.

## Author contributions

All authors listed have made a substantial, direct, and intellectual contribution to the work and approved it for publication.

